# Animal products, calcium and protein and prostate cancer risk in the Netherlands Cohort Study

**DOI:** 10.1038/sj.bjc.6690472

**Published:** 1999-06

**Authors:** A G Schuurman, P A van den Brandt, E Dorant, R A Goldbohm

**Affiliations:** Department of Epidemiology, Maastricht University, PO Box 616, 6200 MD Maastricht, The Netherlands; Department of Consumer Research and Epidemiology, TNO Nutrition and Food Research Institute, PO Box 360, 3700 AJ Zeist, The Netherlands

**Keywords:** prostate neoplasms, cohort study, meat, dairy, calcium, protein

## Abstract

Prostate cancer risk in relation to consumption of animal products, and intake of calcium and protein was investigated in the Netherlands Cohort Study. At baseline in 1986, 58 279 men aged 55–69 years completed a self-administered 150-item food frequency questionnaire and a questionnaire on other risk factors for cancer. After 6.3 years of follow-up, 642 prostate cancer cases were available for analysis. In multivariate case-cohort analyses adjusted for age, family history of prostate cancer and socioeconomic status, no associations were found for consumption of fresh meat, fish, cheese and eggs. Positive trends in risk were found for consumption of cured meat and milk products (*P*-values 0.04 and 0.02 respectively). For calcium and protein intake, no associations were observed. The hypothesis that dietary factors might be more strongly related to advanced prostate tumours could not be confirmed in our study. We conclude that, in this study, animal products are not strongly related to prostate cancer risk. © 1999 Cancer Research Campaign


*British Journal of Cancer* (1999) **80**(7), 1107–1113
© 1999 Cancer Research Campaign
Article no. bjoc.1999.0472

### Animal products, calcium and protein and prostate cancer risk in the Netherlands Cohort Study

**AG Schuurman[1], PA van den Brandt[1], E Dorant[1] and RA Goldbohm[2]**

[1]Department of Epidemiology, Maastricht University, PO Box 616, 6200 MD Maastricht, The Netherlands; [2]Department of Consumer Research and Epidemiology, TNO Nutrition and Food Research Institute, PO Box 360, 3700 AJ Zeist, The Netherlands

**Summary** Prostate cancer risk in relation to consumption of animal products, and intake of calcium and protein was investigated in the Netherlands Cohort Study. At baseline in 1986, 58 279 men aged 55–69 years completed a self-administered 150-item food frequency questionnaire and a questionnaire on other risk factors for cancer. After 6.3 years of follow-up, 642 prostate cancer cases were available for analysis. In multivariate case-cohort analyses adjusted for age, family history of prostate cancer and socioeconomic status, no associations were found for consumption of fresh meat, fish, cheese and eggs. Positive trends in risk were found for consumption of cured meat and milk products (*P*-values 0.04 and 0.02 respectively). For calcium and protein intake, no associations were observed. The hypothesis that dietary factors might be more strongly related to advanced prostate tumours could not be confirmed in our study. We conclude that, in this study, animal products are not strongly related to prostate cancer risk.

**Keywords:** prostate neoplasms; cohort study; meat; dairy; calcium; protein

Remarkable geographic variation exists in clinical prostate cancer incidence. Annual age-adjusted incidence rates of approximately 1 per 100 000 are found in China and up to 102 per 100 000 in USblacks. Rates for whites in the US vary from about 45 to 65 per 100 000. In Western Europe most incidence rates are around 20–30 per 100 000 although some variation exists (Parkin et al, 1992). The prevalence of latent prostatic carcinomas is estimated to be similar in areas with high and with low total prostate cancer incidence rates (Pienta and Esper, 1993; Boyle et al, 1995). Because of these variations in incidence rates worldwide, environmental factors, particularly dietary factors, are widely considered to be related to prostate cancer risks (Mettlin, 1997). Consumption of animal products such as meat, fish, milk, dairy products and eggs differs between countries with high and low prostate cancer incidence rates and may, therefore, be an explanation for the observed differences in incidence rates.

Results from several cohort (Snowdon et al, 1984; Mills et al, 1989; Severson et al, 1989; Thompson et al, 1989; Hirayama, 1990; Hsing et al, 1990; Gann et al, 1994; Le Marchand et al, 1994; Giovannucci et al, 1995; Gronberg et al, 1996) and case-control studies (Schuman et al, 1982; Graham et al, 1983; Mishina et al, 1985; Talamini et al, 1986, 1992; Ross et al, 1987; Oishi et al, 1988; Mettlin et al, 1989; Bravo et al, 1991; Walker et al, 1992; Andersson et al, 1995; Ewings and Bowie, 1996; Pawlega et al, 1996; Key et al, 1997) are available on consumption of animal products and prostate cancer risk. However, the role of animal products remains unclear since contradictory results from both types of studies have been reported.

The effect of animal products on prostate cancer risk is often attributed to their fat content (Pienta and Esper, 1993; Kolonel, 1996; Boyle and Zaridze, 1993; Giles and Ireland, 1997). Increased fat intake might lead to increased testosterone levels and this might, eventually, lead to increased cell division and activation of proto-oncogenes and deactivation of tumour suppressor genes (Ross and Henderson, 1994). However, there is no conclusive evidence on the role of fat in prostate cancer aetiology (Giles and Ireland, 1997). Other hypotheses on the mechanism of action, therefore, deserve consideration, such as that $1,25(OH)_2D$, which is a vitamin D metabolite, levels are protective (Corder et al, 1993, 1995) and calcium intake of which dairy products are a major dietary source, may increase prostate cancer risk by suppressing $1,25(OH)_2D$ levels (Giovannucci et al, 1998). Another possibility is that mutagenic heterocyclic amines, produced when meat is burned at high temperatures, may be carcinogenic (Felton et al, 1997). It has been suggested that dietary factors may play a greater role in accelerating tumour growth than in initiating cancer (Kolonel, 1996; Giles and Ireland, 1997; Mettlin, 1997) but thus far it is not clear whether this is true for animal products. We have investigated animal products consumption as well as calcium intake in relation to prostate cancer risk in the Netherlands Cohort Study (NLCS); intake of protein (total, animal and vegetable) was also evaluated.

### MATERIALS AND METHODS

### The cohort

The NLCS was initiated in September 1986 and has been described in detail elsewhere (Van den Brandt et al., 1990a). The male cohort consists of 58 279 men aged 55–69 years who completed a self-administered questionnaire on usual diet, and other risk factors for cancer. The case-cohort approach (Prentice, 1986) was used in which cases are derived from the entire cohort (providing numerator information for calculation of cancer incidence rates), while accumulated person years at risk in the total cohort are estimated using a random subcohort sample (providing

*Received 16 September 1998*
*Revised 15 December 1998*
*Accepted 4 January 1999*

*Correspondence to:* AG Schuurman denominator information for the rates). A subcohort was sampled from the total cohort and consists of 1688 men. In the follow-up for cancer described previously (Van den Brandt et al, 1990*b*), incident prostate cancer cases were detected by computerized record linkage with all nine cancer registries in The Netherlands, and with the Dutch national data base of pathology reports (PALGA). The subcohort has been followed up biennially for vital status information. Completeness of cancer follow-up was at least 96% (Goldbohm et al, 1994*b*) and follow-up of person years in the subcohort was complete. After a follow-up period of 6.3 years (September 1986–December 1992), 704 incident, microscopically confirmed, primary prostate cancer cases were detected. During this period, systematic screening for prostate cancer was not used in The Netherlands.

### The questionnaire

Usual consumption of food and beverages during the year preceding the start of the study was assessed with a 150-item semi-quantitative food frequency questionnaire (Goldbohm et al, 1994*a*) including 14 different individual fresh meat items (several cuts of beef and pork, minced meat, chicken, liver, other meat), fish, 14 milk and milk items (whole, low-fat and skimmed milk, cream, buttermilk, chocolate milk, dry curd, whole and skimmed yogurt, other items) and eggs. For fresh meat items, participants also had to indicate their usual amount of consumption in grams (as bought, i.e. based on raw meat). For four cured meat items (boiled ham, bacon, lean meat products including smoked beef, and 'other sliced cold meats') and two cheese items (fat cheese and low-fat cheese) subjects had to indicate how many slices of bread they ate with the particular product on it. For other items, subjects had to indicate the consumption amount in natural or household units (e.g. glass). Mean daily consumption (g per day) of the items was calculated by multiplying frequency of consumption by amount of consumption with standard portion sizes for the items that were asked in natural or household units. Calcium and protein intake were computed using the computerized Dutch food composition table (Nevo Tabel, 1986). The questionnaire has been validated against a 9-day diet record. For the exposures under study the Spearman correlation coefficients between questionnaire and the dietary record were as follows: fresh meat 0.46, cured meat 0.54; milk and milk products 0.60; cheese 0.61; fish 0.53; eggs 0.61. The Pearson correlation coefficient (energy and sex-adjusted) for calcium was 0.62 and for total protein, vegetable protein and animal protein the estimates were 0.59, 0.68 and 0.64 respectively (Goldbohm et al, 1994*a*).

### Data analysis

Subjects who reported a history of cancer at baseline, other than skin cancer, were excluded. Furthermore, our criteria (Goldbohm et al, 1994*a*), required exclusion of subjects with incomplete or inconsistent dietary data; 642 men with prostate cancer and 1525 male subcohort members remained for analysis.

Intake of calcium and protein was adjusted for energy by regression analysis (Willett, 1990). Mean intake levels of the different exposure variables and other characteristics were compared between prostate cancer cases and male subcohort members. Furthermore, mean intakes of fresh meat, fish, cured meat, milk and milk products, cheese, eggs, calcium and protein were compared in categories of potential confounders, namely age, a family history of prostate cancer and socioeconomic status. Total energy and total fat intake were not considered as potential confounding factors because no association with prostate cancer risk was observed in our study (data not shown). The same applies to vegetable and fruit consumption (Schuurman et al, 1998). Energy was, however, included in the analyses for calcium and protein, whereas total protein was included in the analyses of animal and vegetable protein to assess the substitution effects of the two sources of protein. Rate ratios (RR) and 95% confidence intervals (95% CI) were computed for quintiles or categories of exposure variables, as well as for continuous variables, using the GLIM statistical package (Baker, 1985). Exponentially distributed survival times were assumed in the follow-up period. Since standard software was not available, specific macros were developed to account for the additional variance introduced by using the subcohort instead of the entire cohort (Volovics and van den Brandt, 1997). Tests for trend were based on likelihood ratio tests. Throughout this report two-sided *P*-values are used. Age-adjusted and multivariate analyses were conducted. In order to evaluate the independent contribution of each specific type of fresh meat, cured meat and dairy items analyses were done with the inclusion of total fresh meat, total cured meat and total dairy consumption, respectively, in the multivariate models. Furthermore, analyses were done for localized (T0–2, M0) and advanced (T3–4, M0; T0–4, M1) prostate cancer cases separately. This classification is based on the TNM staging system. To evaluate whether preclinical symptoms may have influenced results, additional analyses with exclusion of cases detected during the first 2 years of follow-up were conducted.

**Table 1** Description of mean daily intake of animal products, protein and calcium and other characteristics in prostate cancer cases and subcohort members, Netherlands Cohort Study (1986–1992)

| Characteristics | Cases ($n$ = 642) mean (s.d.) | Subcohort ($n$ = 1525) mean (s.d.) |
|---|---|---|
| *Exposure variables (g per day)* | | |
| Fresh meat and poultry[a] | 102.9 (39.6) | 105.2 (43.1) |
| Fish | 15.0 (17.2) | 14.2 (16.0) |
| Cured meat[b] | 15.2 (15.0) | 15.7 (17.3) |
| Milk and milk products[c] | 307.1 (190.1) | 308.0 (215.0) |
| Cheese[d] | 23.0 (18.7) | 22.8 (19.4) |
| Eggs | 16.5 (12.1) | 17.1 (12.5) |
| Calcium (mg per day)[e] | 951.7 (274.7) | 943.7 (292.4) |
| Total protein[e] | 75.3 (10.9) | 75.4 (11.4) |
| Vegetable protein[e] | 27.6 (6.0) | 27.9 (6.0) |
| Animal protein[e] | 48.2 (11.1) | 48.1 (11.8) |
| *Potential confounding variables* | | |
| Age (years) | 63.9 (3.8) | 61.4 (4.2) |
| Family history of prostate cancer (% yes) | 4.4 | 2.7 |
| Highest educational level (%)[f] | | |
| Low | 44.4 | 46.8 |
| Medium | 34.6 | 34.8 |
| High | 20.2 | 17.8 |

[a]This includes beef, pork, minced meat (beef and pork), poultry, liver and 'other meat' (raw weight). [b]This includes boiled ham, bacon, lean meat products (including smoked beef) and 'other sliced cold meats' (several types of sausages). [c]This includes fermented milk products, and non-fermented milk products. [d]This includes fat cheese and low-fat cheese. [e]Energy-adjusted. [f]There was missing information for 0.8% (cases) and 0.7% (subcohort members); low is defined as primary school with/without lower level vocational education, medium as secondary school or medium level vocational education, high as university or higher level vocational education.

© 1999 Cancer Research Campaign

**Table 2** Rate ratios (RRs) and 95% confidence intervals (95% CI) for prostate cancer according to quintiles or categories of consumption of meat, fish, processed meat, milk and dairy, cheese and eggs, Netherlands Cohort Study (1986–1992)

| Exposure | Q1[a] | Q2 | Q3 | Q4 | Q5 | *P* for trend |
|---|---|---|---|---|---|---|
| Fresh meat and poultry | | | | | | |
| Median intake[b] | 56 | 85 | 102 | 123 | 158 | |
| Cases/Person years | 128/1820 | 138/1919 | 144/1787 | 121/1766 | 111/1830 | |
| RR (95% CI)[c] | 1.00 | 1.08 (0.80–1.46) | 1.23 (0.91–1.67) | 1.05 (0.77–1.43) | 1.01 (0.73–1.39) | 0.98 |
| RR (95% CI)[d] | 1.00 | 1.08 (0.79–1.47) | 1.27 (0.93–1.73) | 1.12 (0.82–1.53) | 1.07 (0.77–1.47) | 0.52 |
| Fish | | | | | | |
| Median intake[b,e] | 0 | 5 | 14 | 32 | | |
| Cases/Person years | 162/2238 | 135/2115 | 119/1874 | 226/2895 | | |
| RR (95% CI)[c] | 1.00 | 0.85 (0.64–1.13) | 0.95 (0.70–1.28) | 1.06 (0.82–1.37) | | 0.32 |
| RR (95% CI)[d] | 1.00 | 0.83 (0.62–1.11) | 0.95 (0.70–1.29) | 1.03 (0.80–1.34) | | 0.41 |
| Cured meat | | | | | | |
| Median intake[b] | 0 | 5 | 11 | 19 | 36 | |
| Cases/Person years | 118/1849 | 137/1907 | 140/1703 | 124/1880 | 123/1783 | |
| RR (95% CI)[c] | 1.00 | 1.18 (0.87–1.61) | 1.46 (1.07–1.99) | 1.14 (0.84–1.57) | 1.32 (0.96–1.81) | 0.07 |
| RR (95% CI)[d] | 1.00 | 1.22 (0.90–1.67) | 1.50 (1.09–2.06) | 1.18 (0.86–1.62) | 1.37 (1.00–1.89) | 0.04 |
| Milk and milk products | | | | | | |
| Median intake[b] | 74 | 179 | 271 | 376 | 566 | |
| Cases/Person years | 114/1860 | 112/1835 | 132/1803 | 172/1824 | 112/1800 | |
| RR (95% CI)[c] | 1.00 | 1.08 (0.79–1.49) | 1.22 (0.89–1.67) | 1.60 (1.19–2.17) | 1.09 (0.79–1.50) | 0.03 |
| RR (95% CI)[d] | 1.00 | 1.11 (0.80–1.53) | 1.25 (0.91–1.71) | 1.63 (1.20–2.20) | 1.12 (0.81–1.56) | 0.02 |
| Cheese | | | | | | |
| Median intake[b] | 2 | 13 | 19 | 27 | 43 | |
| Cases/Person years | 140/2261 | 121/1620 | 119/1607 | 175/2371 | 87/1264 | |
| RR (95% CI)[c] | 1.00 | 1.29 (0.95–1.74) | 1.16 (0.86–1.58) | 1.34 (1.01–1.77) | 1.26 (0.90–1.76) | 0.04 |
| RR (95% CI)[d] | 1.00 | 1.27 (0.94–1.73) | 1.15 (0.84–1.56) | 1.28 (0.97–1.70) | 1.21 (0.87–1.70) | 0.09 |
| Eggs | | | | | | |
| Median intake[b,e] | 5 | 14 | 29 | | | |
| Cases/Person years | 197/2530 | 208/3243 | 237/3349 | | | |
| RR (95% CI)[c] | 1.00 | 0.90 (0.70–1.15) | 0.96 (0.76–1.22) | | | 0.72 |
| RR (95% CI)[d] | 1.00 | 0.89 (0.69–1.14) | 0.96 (0.75–1.22) | | | 0.71 |

[a]Reference category. [b]Median intake (g per day) in subcohort; cut-points fresh meat and poultry 73, 93, 108, 137; fish 0, 10, 20; cured meat 2, 8, 14, 25; milk and milk products 139, 220, 321, 460; cheese 7, 14, 21, 37; eggs 7, 14. [c]Adjusted for age. [d]Adjusted for age, family history of prostate cancer and socioeconomic status. [e]Categories instead of quintiles.

### RESULTS

The mean intake of fresh meat, fish, cured meat, milk and milk products, cheese, eggs, calcium and protein among cases and subcohort members is shown in Table 1. None of these food products differed markedly between cases and subcohort members in main in the Table. The distribution of potential confounding factors is also shown in Table 1. Cases are older than subcohort members and more often have a positive family history of prostate cancer. Furthermore, cases more often have a high socioeconomic status compared to subcohort members. Consumption of cured meat was highest among the youngest men and protein intake was highest among men aged 60–64 years. Men in the lowest category of socioeconomic status consumed more fresh meat, cured meat and eggs, and less cheese. Men with a positive family history of prostate cancer consumed more calcium than men without such a family history. Mean consumption of animal products or protein intake differed not between subjects with and without a family history of prostate cancer (data not shown).

RRs for quintile or categorized variables for clusters of food items are shown in Table 2. For total fresh meat and fish consumption no associations with prostate cancer were observed. For both total cured meat and milk and milk products a positive trend in risk was observed (*P*-values for trend test were 0.04 and 0.02 respectively). The RRs (95% CI) were 1.37 (1.00–1.89) for cured meat and 1.12 (0.81–1.56) for milk and milk products for the highest versus lowest quintile of consumption. Only the RR in the fourth quintile of consumption of milk and milk products was significantly increased (RR = 1.63, 95% CI 1.20–2.20). In the age-adjusted analysis, consumption of cheese showed a positive trend in risk ($P = 0.04$), this *P*-value was 0.09 in the multivariate analysis. The RR for the highest versus the lowest category of consumption was 1.21 (95% Cl 0.87–1.70). Egg consumption showed no association with prostate cancer risk. After exclusion of cases diagnosed in the first 2 years of follow-up, RRs were virtually the same.

Table 3 shows results for animal products evaluated as continuous variables, for all tumours and separately for localized and advanced prostate tumours. Within the cluster of fresh meat and poultry items, none of the continuous variables was clearly associated with risk of prostate cancer. Also in subgroups of localized and advanced prostate tumours mostly no association existed. Only for consumption of liver an inverse association with advanced prostate tumours was observed (RR per 5 g increment = 0.79, 95% CI 0.63–0.99). An item on horsemeat, lamb and mutton and an item on consumption of veal were included in the other meat category. The RR (95% CI) per 5 g for consumption of

© 1999Cancer Research Campaign 

**Table 3** Rate ratios (RRs) and 95% confidence intervals (95% CI) for prostate cancer for continuous variables of consumption of animal products, for all cases and separately for localized (T0–2, M0) and advanced (T3–4, M0; T0–4, M1) tumours, Netherlands Cohort Study (1986–1992)

| Exposure | Intake in subcohort (g per day) Mean (s.d.) | Increment | All tumours (*n* = 642) RR (95% CI)[a] | Localized tumours (*n* = 226) RR (95% CI)[a] | Advanced tumours (*n* = 213) RR (95% CI)[a] |
|---|---|---|---|---|---|
| Fresh meat and poultry | 105.2 (43.1) | 25 | 1.00 (0.86–1.16) | 0.99 (0.91–1.08) | 1.00 (0.91–1.09) |
| Beef[b] | 27.4 (23.6) | 25 | 1.00 (0.89–1.12) | 0.95 (0.80–1.12) | 0.92 (0.77–1.10) |
| Pork[b] | 40.6 (31.1) | 25 | 1.06 (0.96–1.18) | 1.16 (1.00–1.34) | 1.06 (0.91–1.23) |
| Minced meat (beef and pork)[b] | 20.0 (19.0) | 25 | 0.86 (0.74–1.01) | 0.84 (0.66–1.07) | 0.90 (0.71–1.14) |
| Chicken[b] | 13.4 (15.0) | 25 | 1.00 (0.84–1.18) | 0.89 (0.68–1.16) | 1.11 (0.87–1.42) |
| Liver[b] | 2.1 (4.7) | 5 | 0.92 (0.82–1.04) | 0.99 (0.85–1.17) | 0.79 (0.63–0.99) |
| Other meat[b] | 2.8 (6.0) | 5 | 1.06 (0.99–1.15) | 1.04 (0.93–1.16) | 1.09 (0.98–1.21) |
| Fish | 14.2 (16.0) | 25 | 1.06 (0.91–1.22) | 0.91 (0.73–1.15) | 1.08 (0.87–1.33) |
| Cured meat | 15.7 (17.3) | 15 | 1.03 (0.94–1.12) | 1.03 (0.91–1.17) | 1.00 (0.88–1.14) |
| Boiled ham[c] | 5.4 (8.1) | 15 | 0.94 (0.73–1.21) | 1.00 (0.69–1.45) | 0.95 (0.64–1.40) |
| Bacon[c] | 2.0 (5.1) | 15 | 0.80 (0.57–1.13) | 0.79 (0.47–1.33) | 1.04 (0.66–1.65) |
| Lean meat products[c] | 2.4 (5.4) | 15 | 0.93 (0.68–1.27) | 0.78 (0.48–1.29) | 1.01 (0.63–1.60) |
| Other sliced cold meat[c] | 6.0 (10.2) | 15 | 1.18 (0.96–1.44) | 1.22 (0.90–1.65) | 1.01 (0.74–1.39) |
| Milk and milk products | 308.0 (215.0) | 50 | 1.00 (0.98–1.03) | 1.01 (0.98–1.05) | 0.99 (0.95–1.03) |
| Whole milk, fermented[d] | 15.9 (40.1) | 50 | 0.87 (0.76–1.00) | 0.96 (0.79–1.15) | 0.84 (0.66–1.05) |
| Low-fat milk, fermented[d] | 68.4 (107.5) | 50 | 1.01 (0.96–1.07) | 1.01 (0.94–1.09) | 1.03 (0.95–1.11) |
| Whole milk[d] | 136.0 (164.4) | 50 | 1.00 (0.96–1.03) | 0.97 (0.92–1.03) | 1.00 (0.95–1.06) |
| Low-fat milk[d] | 87.8 (139.4) | 50 | 1.01 (0.97–1.05) | 1.03 (0.97–1.09) | 0.99 (0.93–1.06) |
| Cheese | 22.8 (19.4) | 20 | 1.02 (0.93–1.13) | 1.20 (1.06–1.37) | 0.92 (0.78–1.08) |
| Cheese[e] | 21.1 (18.6) | 20 | 0.99 (0.76–1.30) | 0.94 (0.68–1.29) | 1.05 (0.66–1.68) |
| Low-fat cheese[e] | 1.7 (7.6) | 20 | 1.01 (0.77–1.32) | 1.07 (0.78–1.47) | 0.95 (0.60–1.52) |
| Eggs | 17.1 (12.5) | 20 | 0.95 (0.81–1.11) | 0.99 (0.78–1.24) | 0.70 (0.53–0.93) |

[a]Adjusted for age, family history of prostate cancer, and socioeconomic status. [b]Additional adjustment for consumption of total fresh meat and poultry. [c]Additional adjustment for consumption of total cured meat. [d]Additional adjustment for consumption of total milk and milk products. [e]Additional adjustment for consumption of total cheese.

horsemeat, lamb and mutton was 1.11 (1.02–1.21) and 1.07 (0.99–1.15) for consumption of veal (data not shown). Total fish and total cured meat consumption were not associated with overall prostate cancer risk, or with risk of localized and advanced tumours. Evaluated as continuous variables, none of the individual cured meat items was strongly related to prostate cancer risk. Nevertheless, in categorized analyses a positive trend in risk was noted for consumption of ‘other sliced cold meats’ ($P$-value trend test = 0.02). For the highest versus the lowest consumption category a RR of 1.37 (95% CI 1.03–1.83) was found in the multivariate analysis. This increase in risk was only found in the subgroup of localized prostate tumours (RR for the same contrast = 1.44, 95% CI 0.95–2.20) and not for advanced prostate tumours (data not shown). Fermented whole milk showed a borderline significant inverse association with overall prostate cancer risk (RR per 50 g = 0.87, 95% CI 0.76–1.00), and also for advanced tumours an inverse association was suggested (RR per 50 g = 0.84, 95% CI 0.66–1.05). For none of the individual milk items except consumption of whole yoghurt, associations with prostate cancer risk were observed. In the continuous model, the RR for consumption of whole yoghurt per 50 g increment was 0.88 (95% CI 0.76–1.01). Cheese consumption showed no association with overall prostate cancer risk, but was positively associated with localized prostate tumours (RR per 20 g = 1.20, 95% CI 1.06–1.37). Finally, consumption of eggs was inversely associated with advanced prostate tumours (RR per 20 g = 0.70, 95% CI 0.53–0.93).

The results for calcium and protein intake are shown in Table 4. For intake of calcium and total protein no associations with prostate cancer risk were noted. Controlling for total protein, intake of vegetable protein showed RR below one in all four categories, but none of the RRs was statistically significant. All RRs for intake of animal protein were above one. Only the RR in the fourth quintile of intake was statistically significant (RR = 1.52, 95% CI 1.01–2.30). For both sources of protein intake, no trend in risk was found. We also examined calcium and protein intake in subgroups of localized and advanced prostate tumours. As for the animal products, there was no clear tendency for stronger associations with advanced prostate tumours.

### DISCUSSION

Overall consumption of fresh meat and poultry, fish, cheese and eggs showed no association with prostate cancer risk in the NLCS. The observed positive trend in risk for quintiles of total cured meat consumption could be explained by a positive association with consumption of ‘other sliced cold meats’. For most clusters of milk items, or individual milk items, no strong associations were observed, but consumption of whole yoghurt might be associated with a decreased prostate cancer risk. Intake of calcium and protein was not associated with risk of prostate cancer in our study. Finally, we found no clear evidence of a stronger association of various products with advanced prostate cancer.

The NLCS is a prospective cohort study specifically designed to evaluate the relation between diet and cancer. An important strength of prospective studies is that recall bias is avoided because of the prospective nature of these studies. Selection bias is also not likely to have taken place because of the high completeness of follow-up of subcohort members (Van den Brandt et al, 1993; Goldbohm et al, 1994*b*). A 150-item semi-quantitative food

 *© 1999 Cancer Research Campaign*

**Table 4** Rate ratios (RRs) and 95% confidence intervals (95% CI) for prostate cancer according to quintiles of intake of energy-adjusted calcium and energy-adjusted protein, for all cases and separately for localized (T0–2, M0) and advanced (T3–4, M0; T0–4, M1) prostate tumours, Netherlands Cohort Study, 6.3 years of follow-up (1986–1992)

| Exposure | Q1[a] | Q2 | Q3 | Q4 | Q5 | *P* for trend |
|---|---|---|---|---|---|---|
| Calcium | | | | | | |
| Median intake (mg per day)[b] | 602 | 780 | 911 | 1064 | 1329 | |
| Cases/Person years | 120/1821 | 126/1845 | 127/1840 | 140/1817 | 129/1800 | |
| RR (95% CI)[c] | 1.00 | 1.07 (0.79–1.47) | 1.03 (0.75–1.41) | 1.20 (0.88–1.64) | 1.07 (0.79–1.47) | 0.36 |
| RR (95% CI)[d] | 1.00 | 1.10 (0.80–1.51) | 1.04 (0.76–1.42) | 1.21 (0.89–1.66) | 1.09 (0.79–1.50) | 0.34 |
| Localized tumours (*n*) | 47 | 30 | 45 | 46 | 56 | |
| RR (95% CI)[d] | 1.00 | 0.69 (0.42–1.13) | 0.96 (0.61–1.50) | 1.04 (0.67–1.63) | 1.21 (0.79–1.86) | 0.10 |
| Advanced tumours (*n*) | 44 | 46 | 37 | 46 | 37 | |
| RR (95% CI)[d] | 1.00 | 1.08 (0.69–1.70) | 0.79 (0.49–1.27) | 1.06 (0.67–1.66) | 0.83 (0.52–1.34) | 0.45 |
| Total protein | | | | | | |
| Median intake (g per day)[b] | 62 | 69 | 75 | 81 | 90 | |
| Cases/Person years | 128/1839 | 121/1836 | 134/1821 | 135/1792 | 124/1834 | |
| RR (95% CI)[c] | 1.00 | 1.03 (0.75–1.41) | 1.10 (0.81–1.50) | 1.31 (0.96–1.79) | 1.09 (0.80–1.48) | 0.15 |
| RR (95% CI)[d] | 1.00 | 1.04 (0.76–1.43) | 1.12 (0.82–1.53) | 1.35 (0.98–1.84) | 1.10 (0.81–1.51) | 0.11 |
| Localized tumours (*n*) | 51 | 34 | 40 | 49 | 50 | |
| RR (95% CI)[d] | 1.00 | 0.76 (0.47–1.22) | 0.87 (0.55–1.38) | 1.27 (0.82–1.96) | 1.13 (0.73–1.74) | 0.13 |
| Advanced tumours (*n*) | 46 | 43 | 48 | 38 | 35 | |
| RR (95% CI)[d] | 1.00 | 1.04 (0.66–1.64) | 1.08 (0.69–1.69) | 1.02 (0.64–1.64) | 0.83 (0.51–1.33) | 0.49 |
| Vegetable protein | | | | | | |
| Median intake (g per day)[b] | 22 | 25 | 27 | 30 | 35 | |
| Cases/Person years | 143/1827 | 129/1833 | 139/1813 | 110/1812 | 121/1839 | |
| RR (95% CI)[c] | 1.00 | 0.87 (0.64–1.18) | 0.99 (0.74–1.34) | 0.83 (0.61–1.13) | 0.92 (0.67–1.24) | 0.43 |
| RR (95% CI)[e] | 1.00 | 0.86 (0.63–1.17) | 1.00 (0.74–1.36) | 0.83 (0.61–1.14) | 0.90 (0.66–1.23) | 0.37 |
| Localized tumours (*n*) | 53 | 46 | 42 | 35 | 48 | |
| RR (95% CI)[e] | 1.00 | 0.86 (0.55–1.33) | 0.85 (0.54–1.33) | 0.73 (0.46–1.17) | 0.96 (0.62–1.48) | 0.63 |
| Advanced tumours (*n*) | 38 | 43 | 57 | 30 | 42 | |
| RR (95% CI)[e] | 1.00 | 1.08 (0.67–1.74) | 1.55 (0.98–2.44) | 0.85 (0.51–1.43) | 1.19 (0.74–1.92) | 0.81 |
| Animal protein | | | | | | |
| Median intake (g per day)[b] | 34 | 42 | 47 | 53 | 64 | |
| Cases/Person years | 112/1825 | 137/1843 | 121/1819 | 150/1812 | 122/1823 | |
| RR (95% CI)[c] | 1.00 | 1.21 (0.88–1.65) | 1.11 (0.81–1.53) | 1.42 (1.04–1.93) | 1.16 (0.84–1.59) | 0.11 |
| RR (95% CI)[e] | 1.00 | 1.29 (0.92–1.81) | 1.16 (0.80–1.68) | 1.52 (1.01–2.30) | 1.32 (0.76–2.29) | 0.09 |
| Localized tumours (*n*) | 44 | 40 | 44 | 45 | 51 | |
| RR (95% CI)[e] | 1.00 | 0.94 (0.57–1.55) | 1.08 (0.64–1.83) | 1.13 (0.62–2.05) | 1.26 (0.58–2.75) | 0.44 |
| Advanced tumours (*n*) | 43 | 49 | 41 | 47 | 30 | |
| RR (95% CI)[e] | 1.00 | 1.21 (0.75–1.95) | 0.96 (0.56–1.65) | 1.11 (0.61–2.04) | 0.71 (0.31–1.63) | 0.61 |

[a]Reference category. [b]Median intake in subcohort; cut-points calcium 709, 848, 984, 1164; total protein 66, 72, 77, 84; vegetable protein 23, 26, 29, 32; animal protein 38, 45, 50, 58. [c]Adjusted for age. [d]Adjusted for age, family history of prostate cancer, socioeconomic status and total energy intake. [e]Adjusted for age, family history of prostate cancer, socioeconomic status, total energy intake and total protein intake.

frequency questionnaire was used to estimate the usual consumption of fresh meat and poultry, fish, cured meat, milk and milk products, cheese and eggs during the year preceding the start of the study. The questionnaire was validated against a 9-day dietary record. Based on the Spearman correlation coefficients we conclude that our exposure variables were reasonably well measured. In addition, these correlation coefficients may be underestimated because many of the record data were coded as ingredients from recipes or mixed dishes as opposed to the questionnaire data, which were coded as food product. Consequently, the division between food groups was not always clear, resulting in lower correlations (Goldbohm et al, 1994*a*). Misclassification of subjects according to their exposure status is possible, but expected to be non-differential. To prevent substantial misclassification of subjects with respect to exposure status, subjects with incomplete or inconsistent data were excluded, according to criteria published before (Goldbohm et al, 1994*a*). Besides a validation study, five annually repeated measurements of the food frequency questionnaire were conducted. From the results it was concluded that the single measurement of diet in the NLCS can characterize dietary habits for a period of at least 5 years (Goldbohm et al, 1995). This is further supported by the fact that our study population consists of older subjects (aged 55–69 years) with relatively stable dietary habits (Van den Brandt et al, 1990*a*).

Data gathered with our questionnaire allowed us to take other dietary and non-dietary risk factors for prostate cancer into account in multivariate analyses. Although our final multivariate model was also somewhat restricted, we considered several potential confounding factors and only those factors associated with prostate cancer risk in our study were included in the model. Certainly, unmeasured or still unknown other factors may have caused residual confounding. Results after exclusion of cases detected in the first 2 years of follow-up were similar to those that included all prostate cancer cases. Therefore, preclinical disease is not likely to have influenced our results. Finally, chance will have played a role in our study, in particular because of the multiple associations that were studied.

Only a minority of previous cohort (Snowdon et al, 1984; Giovannucci et al, 1993, 1995) and case-control studies (Mettlin et al, 1989; Talamini et al, 1992; Andersson et al, 1995) had a fairly

© 1999Cancer Research Campaign 

comprehensive measurement of dietary habits. Therefore (random) misclassification of exposure may have affected results in earlier studies. Furthermore, results from most other studies were based on substantially less cases than the total number of cases in our study. There were only two cohort studies with more than 400 cases (Giovannucci et al, 1993, 1995; Gronberg et al, 1996) and only three case-control studies with more than 300 cases (Graham et al, 1983; Mettlin et al, 1989; Key et al, 1997). Comparisons of different studies is also hampered by the fact that endpoints in previous studies were either incidence or mortality. Deceased prostate cancer cases may not adequately reflect the source population of total prostate cancer cases. Finally, limited adjustment for confounding factors may have influenced results in different studies.

Total meat consumption or consumption of specific types of meat were not clearly associated with prostate cancer risk in several other cohort studies (Snowdon et al, 1984; Mills et al, 1989; (Severson et al, 1989; Hsing et al, 1990; Giovannucci et al, 1995) and case-control studies (Schuman et al, 1982; Talamini et al, 1992; Andersson et al, 1995; Key et al, 1997). On the other hand, positive associations were observed, in other cohort studies for consumption of meat (Hirayama, 1990), high fat animal products and beef (Le Marchand et al, 1994), beef, pork and lamb (Giovannucci et al, 1993; Gann et al, 1994), and for meat, poultry and fish (Mills et al, 1989) and in case-control studies for consumption of meat (Mishina et al, 1985; Talamini et al, 1986; Walker et al, 1992), lamb and pork (Bravo et al, 1991) and meat and fish combined (Graham et al, 1983). In other cohort studies inverse associations were suggested for consumption of beef (Gronberg et al, 1996) and bacon or side pork (Schuman et al, 1982), and in case-control studies for consumption of poultry or chicken (Schuman et al, 1982; Ross et al, 1987) and liver (Pawlega et al, 1996).

As in our study, intake of fish was not associated overall with prostate cancer risk in cohort studies (Severson et al, 1989; Hsing et al, 1990; Le Marchand et al, 1994; Gronberg et al, 1996), though a positive (Mills et al, 1989) and an inverse association (Hirayama, 1990) have also been reported. From case-control studies positive (Andersson et al, 1995), inverse (Schuman et al, 1982; Pawlega et al, 1996; Key et al, 1997) and null associations (Talamini et al, 1992) have been recorded.

One cohort study reported on processed meats in relation to prostate cancer risk and in this study no association was found (Le Marchand et al, 1994). Our data suggested a positive association between consumption of ‘other sliced cold meats’ and prostate cancer risk. Although ‘other sliced cold meats’ were not defined further in our questionnaire, several types of sausages are frequently consumed in The Netherlands and these products are most likely to account for the observed association.

In most cohort studies, intake of milk or other dairy products were not clearly associated with prostate cancer risk (Mills et al, 1989; Severson et al, 1989; Thompson et al, 1989; Hirayama, 1990; Hsing et al, 1990; Le Marchand et al, 1994; Giovannucci et al, 1995; Gronberg et al, 1996); in only one cohort study was a positive association reported (Snowdon et al, 1984). From case-control studies on milk or dairy products, however, positive associations were reported more frequently (Mishina et al, 1985; Talamini et al, 1986; Mettlin et al, 1989; Talamini et al, 1992), although in this type of study also null associations have been found (Schuman et al, 1982; Andersson et al, 1995; Ewings and Bowie, 1996). To our knowledge, an (inverse) association between fermented milk products and prostate cancer risk has not been reported elsewhere, although an inverse association has been reported in other hormone-related cancers (Van ‘t Veer et al, 1989).

Consumption of cheese (Snowdon et al, 1984) and cheese in combination with butter and margarine (Severson et al, 1989) were associated with a modest increase in risk, in two cohort studies. In two case-control studies no associations were found (Talamini et al, 1992; Andersson et al, 1995). Egg consumption was not associated with prostate cancer risk in all (Snowdon et al, 1984; Mills et al, 1989 Thompson et al, 1989; Hsing et al, 1990; Le Marchand et al, 1994; Giovannucci et al, 1995; Gronberg et al, 1996) except one cohort study, in which a positive association was indicated (Severson et al, 1989). Results from case-control studies were more diverse, varying from a suggestive inverse association (Ewings and Bowie, 1996), and null associations (Schuman et al, 1982; Talamini et al, 1992; Andersson et al, 1995) to positive associations (Ross et al, 1987; Walker et al, 1992).

As in certain other studies (Le Marchand et al, 1994; Andersson et al, 1995; Giovannucci et al, 1995), we evaluated risk factors separately for localized and advanced tumours, though some 30% of our cases could not be so classified because of missing information on tumour characterization. The results from our and other studies do not uniformly point at stronger associations between the exposure variables and advanced prostate tumours. Because the number of studies in which subgroup analyses based on tumour characterization is low, definite conclusions cannot be drawn yet.

From the results of the NLCS and other studies we conclude that, thus far, there is no convincing evidence for an important role of the consumption of fresh meat, fish, cured meat, milk and milk products, cheese and eggs in prostate cancer aetiology. It has to be mentioned, however, that even the lower tail of the distribution of consumption of animal products in the NLCS and in most of the other studies represents a higher consumption than the average consumption level in countries with low prostate cancer incidence rates. Therefore, the possibility that at much lower levels consumption of animal products is important in prostate cancer aetiology cannot be ruled out.

In our study we could not confirm a positive association between calcium intake and prostate cancer risk, which has recently been proposed (Giovannucci et al, 1998). Furthermore, there were no clear associations as with animal or vegetable protein. More studies are needed to investigate the suggested role of calcium intake in prostate cancer aetiology. Other studies should also evaluate whether a diet based on animal foods might be positively associated with prostate cancer risk and whether plant-based foods might be protective. In future studies, long follow-up periods with repeated extensive measurements of diet could be helpful in evaluating whether diet is involved in prostate cancer progression or whether diet has an effect relatively early in carcinogenesis. Finally, mechanistic research is also warranted.

### ACKNOWLEDGEMENTS

We are indebted to the participants of this study and further wish to thank the regional cancer registries (IKA, IKL, IKMN, IKN, IKO, IKR, IKST, IKW, IKZ), and the Dutch national data base of pathology (PALGA); A Volovics for statistical advice; S van de Crommert, J Nelissen, H Brants, M Moll, W van Dijk, P Florax and A Pisters for assistance; and H van Montfort, R Schmeitz, T van Montfort, and M de Leeuw for programming and statistical assistance. The Netherlands Cohort Study was supported by the Dutch Cancer Society.

*© 1999 Cancer Research Campaign*

**REFERENCES**

Andersson SO, Baron J, Wolk A, Lindgren C, Bergstrom R and Adami HO (1995) Early life risk factors for prostate cancer: a population-based case-control study in Sweden. *Cancer Epidemiol Biomarkers Prev* **4**: 187–192

Baker J (1985) *GLIM 3.77 Reference Manual*. Numerical Algorithms Group: Oxford

Boyle P, Maisonneuve P and Napalkov P (1995) Geographical and temporal patterns of incidence and mortality from prostate cancer. *Urology* **46**: 47–55

Boyle P and Zaridze DG (1993) Risk factors for prostate and testicular cancer. *Eur J Cancer* **29a**: 1048–1055

Bravo MP, Castellanos E and del Rey Calero J (1991) Dietary factors and prostatic cancer. *Urol Int* **46**: 163–166

Corder EH, Friedman GD, Vogelman JH and Orentreich N (1995) Seasonal variation in vitamin D, vitamin D-binding protein, and dehydroepiandrosterone: risk of prostate cancer in black and white men. *Cancer Epidemiol Biomarkers Prev* **4**: 655–659

Corder EH, Guess HA, Hulka BS, Friedman GD, Sadler M, Vollmer RT, Lobaugh B, Drezner MK, Vogelman JH and Orentreich N (1993) Vitamin D and prostate cancer: a prediagnostic study with stored sera. *Cancer Epidemiol Biomarkers Prev* **2**: 467–472

Ewings P and Bowie C (1996) A case-control study of cancer of the prostate in Somerset and east Devon. *Br J Cancer* **74**: 661–666

Felton JS, Malfatti MA, Knize MG, Salmon CP, Hopmans EC and Wu RW (1997) Health risks of heterocyclic amines. *Mutat Res* **376**: 37–41

Gann PH, Hennekens CH, Sacks FM, Grodstein F, Giovannucci EL and Stampfer MJ (1994) Prospective study of plasma fatty acids and risk of prostate cancer. *J Natl Cancer Inst* **86**: 281–286

Giles G and Ireland P (1997) Diet, nutrition and prostate cancer. *Int J Cancer* **Suppl 10**: 13–17

Giovannucci E, Rimm EB, Colditz GA, Stampfer MJ, Ascherio A, Chute CC and Willett WC (1993) A prospective study of dietary fat and risk of prostate cancer. *J Natl Cancer Inst* **85**: 1571–1579

Giovannucci E, Ascherio A, Rimm EB, Stampfer MJ, Colditz GA and Willett WC (1995) Intake of carotenoids and retinol in relation to risk of prostate cancer. *J Natl Cancer Inst* **87**: 1767–1776

Giovannucci E, Rimm EB, Wolk A, Ascherio A, Stampfer MJ, Colditz GA and Willett WC (1998) Calcium and fructose intake in relation to risk of prostate cancer. *Cancer Res* **58**: 442–447

Goldbohm RA, Van den Brandt PA, Brants HAM, Van 't Veer P, AI M, Sturmans F and Hermus RJ (1994*a*). Validation of a dietary questionnaire used in a large-scale prospective cohort study on diet and cancer. *Eur J Clin Nutr* **48**: 253–265

Goldbohm RA, Van den Brandt PA and Dorant E (1994*b*) Estimation of the coverage of Dutch municipalities by cancer registries and PALGA based on hospital discharge data. *Tijdschr Soc Gezondheidsz* **72**: 80–84

Goldbohm RA, Van 't Veer P, Van den Brandt PA, Van 't Hof MA, Brants HAM, Sturmans F and Hermus RJ (1995) Reproducibility of a food frequency questionnaire and stability of dietary habits determined from five annually repeated measurements. *Eur J Clin Nutr* **49**: 420–429

Graham S, Haughey B, Marshall J, Priore R, Byers T, Rzepka T, Mettlin C and Pontes JE (1983) Diet in the epidemiology of carcinoma of the prostate gland. *J Natl Cancer Inst* **70**: 687–692

Gronberg H, Damber L and Damber JE (1996) Total food consumption and body mass index in relation to prostate cancer risk: a case-control study in Sweden with prospectively collected exposure data. *J Urol* **155**: 969–974

Hirayama T (1990) *Life-style and Mortality. A Large-scale Census-based Cohort Study in Japan*. Karger: Basel.

Hsing AW, McLaughlin JK, Schuman LM, Bjelke E, Gridley G, Wacholder S, Chien HT and Blot WJ (1990) Diet, tobacco use, and fatal prostate cancer: results from the Lutheran Brotherhood Cohort Study. *Cancer Res* **50**: 6836–6840

Key TJ, Silcocks PB, Davey GK, Appleby PN and Bishop DT (1997) A case-control study of diet and prostate cancer. *Br J Cancer* **76**: 678–687

Kolonel LN (1996) Nutrition and prostate cancer. *Cancer Causes Control* **7**: 83–94

Le Marchand L, Kolonel LN, Wilkens LR, Myers BC and Hirohata T (1994) Animal fat consumption and prostate cancer: a prospective study in Hawaii. *Epidemiology* **5**: 276–282

Mettlin C (1997) Recent developments in the epidemiology of prostate cancer. *Eur J Cancer* **33**: 340–347

Mettlin C, Selenskas S, Natarajan N and Huben R (1989) Beta-carotene and animal fats and their relationship to prostate cancer risk. A case-control study. *Cancer* **64**: 605–612

Mills PK, Beeson WL, Phillips RL and Fraser GE (1989) Cohort study of diet, lifestyle, and prostate cancer in Adventist men. *Cancer* **64**: 598–604

Mishina T, Watanabe H, Araki H and Nakao M (1985) Epidemiological study of prostatic cancer by matched-pair analysis. *Prostate* **6**: 423–436

Nevo Tabel (1986) *Dutch Food Composition Table 1986–1987*. Voorlichtingsbureau voor de voeding: The Hague.

Oishi K, Okada K, Yoshida O, Yamabe H, Ohno Y, Hayes RB and Schroeder FH (1988) A case-control study of prostatic cancer with reference to dietary habits. *Prostate* **12**: 179–190

Parkin DM, Muir CS, Whelan SL, Gao Y-T, Ferlay J and Powell J (1992) *Cancer Incidence in Five Continents*, Vol. VI. IARC Scientific Publications: Lyon

Pawlega J, Rachtan J and Dyba T (1996) Dietary factors and risk of prostate cancer in Poland. Results of case-control study. *Neoplasma* **43**: 61–63

Pienta KJ and Esper PS (1993) Risk factors for prostate cancer. *Ann Intern Med* **118**: 793–803

Prentice RL (1986) A case-cohort design for epidemiologic cohort studies and disease prevention trials. *Biometrika* **73**: 1–11

Ross RK and Henderson BE (1994) Do diet and androgens alter prostate cancer risk via a common etiologic pathway? *J Natl Cancer Inst* **86**: 252–254

Ross RK, Shimizu H, Paganini Hill A, Honda G and Henderson BE (1987) Case-control studies of prostate cancer in blacks and whites in southern California. *J Natl Cancer Inst* **78**: 869–874

Schuman LM, Mandel JS, Radke A, Seal U and Halberg F (1982) Some selected features of the epidemiology of prostatic cancer: Minneapolis–St Paul, Minnesota case-control study, 1976–1979. In *Trends in Cancer Incidence: Causes and Practical Implications*, Magnus K (ed), pp. 345–354. Hemisphere Publishing Corporation: Washington

Schuurman AG, Goldbohm RA, Dorant E and Van den Brandt PA (1998) Vegetable and fruit consumption and prostate cancer risk: a cohort study in the Netherlands. *Cancer Epidemiol Biom Prev* **7**: 673–680

Severson RK, Nomura AM, Grove JS and Stemmermann GN (1989) A prospective study of demographics, diet, and prostate cancer among men of Japanese ancestry in Hawaii. *Cancer Res* **49**: 1857–1860

Snowdon DA, Phillips RL and Choi W (1984) Diet, obesity, and risk of fatal prostate cancer. *Am J Epidemiol* **120**: 244–250

Talamini R, Franceschi S, La Vecchia C, Serraino D, Barra S and Negri E (1992) Diet and prostatic cancer: a case-control study in northern Italy. *Nutr Cancer* **18**: 277–286

Talamini R, La Vecchia C, Decarli A, Negri E and Franceschi S (1986) Nutrition, social factors and prostatic cancer in a northern Italian population. *Br J Cancer* **53**: 817–821

Thompson MM, Garland C, Barrett-Connor E, Khaw KT, Friedlander NJ and Wingard DL (1989) Heart disease risk factors, diabetes, and prostatic cancer in an adult community. *Am J Epidemiol* **129**: 511–517

Van den Brandt PA, Goldbohm RA, Van 't Veer P, Volovics A, Hermus RJ and Sturmans F (1990*a*) A large-scale prospective cohort study on diet and cancer in The Netherlands. *J Clin Epidemiol* **43**: 285–295

Van den Brandt PA, Schouten LJ, Goldbohm RA, Dorant E and Hunen PM (1990*b*) Development of a record linkage protocol for use in the Dutch Cancer Registry for Epidemiological Research. *Int J Epidemiol* **19**: 553–558

Van den Brandt PA, Van 't Veer P, Goldbohm RA, Dorant E, Volovics A, Hermus RJ and Sturmans F (1993) A prospective cohort study on dietary fat and the risk of postmenopausal breast cancer. *Cancer Res* **53**: 75–82

Van 't Veer P, Dekker JM, Lamers JW, Kok FJ, Schouten EG, Brants HAM, Sturmans F and Hermus RJ (1989) Consumption of fermented milk products and breast cancer: a case-control study in The Netherlands. *Cancer Res* **49**: 4020–4023

Volovics A and Van den Brandt PA (1997) Methods for the analyses of case-cohort studies. *Biom J* **2**: 195–214

Walker AR, Walker BF, Tsotetsi NG, Sebitso C, Siwedi D and Walker AJ (1992) Case-control study of prostate cancer in black patients in Soweto, South Africa. *Br J Cancer* **65**: 438–441

Willett W (1990) Implications of total energy intake for epidemiologic analyses. In *Nutritional Epidemiology*, Willett W (ed), pp. 245–271. Monographs in Epidemiology and Biostatistics. Oxford University Press: New York

*© 1999Cancer Research Campaign*
